# Training-Related Sports Injury Patterns Among Elite Middle and High School Field Hockey Players in Korea

**DOI:** 10.3390/sports13040117

**Published:** 2025-04-14

**Authors:** Minkyung Choi, Kihyuk Lee

**Affiliations:** 1 Department of Sport Culture, Dongguk University, 30, Pildong-ro 1gil, Jung-gu, Seoul 04620, Republic of Korea; junochoi89@naver.com; 2 Data Convergence Team, Seoul National University Bundang Hospital, Seongnam 13605, Republic of Korea

**Keywords:** youth field hockey, incidence of sports injury, injury site, prevention method of injury

## Abstract

This study aimed to analyze the incidence, affected areas, and types of injuries sustained during training among youth field hockey players to identify key characteristics. A survey was conducted with 374 youth field hockey players (236 males, 138 females) from middle and high school teams registered with the Korea Hockey Association in 2024. Data were collected on injury status, training hours, injury sites, and prevention methods. Chi-square tests, independent *t*-tests, and frequency analyses were performed to assess differences in injury incidence based on gender and school level. The analysis revealed no significant gender differences in training-related injury experiences, with 17.2% of male athletes and 10.2% of female athletes reporting injuries during training. Similarly, no significant difference was found between middle and high school athletes, although high school athletes had a significantly lower injury incidence than middle school athletes (1.54 vs. 2.28 per 1000 h, *p* < 0.05). The most common injury sites were the ankle and knee, with muscle injuries being the most frequent among males and ligament injuries among females. Additionally, male and high school athletes more frequently reported engaging in warm-up and cool-down exercises. There was no significant difference in injury rates between genders during training, but high school athletes tended to experience fewer injuries than middle school athletes. Ankle injuries were the most common, with muscle and ligament damage being the primary types of injuries. While warm-up exercises were commonly practiced, further research is needed to determine their actual effectiveness in injury prevention.

## 1. Introduction

Field hockey is a sport played with a hard, fast-moving ball and a stick, consisting of four fifteen-minute quarters [1]. It demands high acceleration and rapid changes in direction from players. To meet these physical demands, high-intensity activities are required. Additionally, the unstable postures during dribbling and shooting maneuvers are known to place significant strain on the spine, thereby increasing the risk of lower back and pelvic injuries [2,3]. Moreover, due to the potential for collisions with sticks and balls, as well as physical contact, the risk of injuries such as bone fractures and dislocations is high, necessitating the use of protective equipment [4,5].

The physical demands of sports like field hockey can lead to injuries due to the interaction of environmental, anatomical, biomechanical, and hormonal factors [6]. Specifically, anatomical differences between genders contribute to a larger Q-angle in female athletes, which places excessive strain on the knee ligaments, increasing the risk of injury [7]. Additionally, females are approximately five times more likely to experience an anterior cruciate ligament injury than males [8]. Therefore, optimizing technical and tactical strategies while ensuring a safe environment is crucial in sports like field hockey. In youth sports settings, understanding the extent and nature of injuries is essential for exploring potential approaches to injury prevention [9,10].

In team sports, the combination of quick reaction abilities and high-level coordination is known to form a crucial foundation for successful performance in competitive situations [11]. These skills require athletes to respond quickly and accurately, making systematic training essential to achieve optimal performance. Furthermore, reaction speed and agility also play a significant role in injury prevention. Effective segment coordination allows athletes to synchronize body movements, enabling precise and efficient motor tasks, which can contribute to reducing the occurrence of injuries [11,12], 051-52.

A study on the epidemiology of injuries among field hockey players in the National Collegiate Athletic Association (NCAA) reported a decrease in the game injury rate by an average of 2.5% annually over a fifteen-year period [4]. However, ankle ligament sprains were frequently identified as a common injury, accounting for 13.7% of game-related injuries and 15.0% of practice-related injuries. The injury rate was higher during games than during practices (7.87 vs. 3.70 injuries per 1000 athlete exposures). Another study indicated that fractures accounted for approximately 15% of field hockey injuries [13]. More recently, Lynall et al. found that injury rates among collegiate female field hockey players were higher during the preseason compared to the regular and postseason (high school: 52.0%, college: 60.7%) [14]. Gradual increases in activity levels during the preseason, coupled with proper conditioning through enhanced offseason programs, may help reduce the risk of injury [15]. While studies on the epidemiology of injuries occurring during the preseason in field hockey are available, there remains a gap in epidemiological investigations concerning injuries during training throughout the entire season.

Additionally, it has been emphasized that early implementation of injury prevention programs is crucial [16], and recording and comparing injury incidences at the high school athlete level is important for effective prevention [14]. However, compared to injury-related data from Western countries, data on injuries among Asian field hockey players remain limited. Furthermore, there is a significant lack of data on elite youth athletes in comparison to adult athletes. This scarcity of data hampers the development of evidence-based injury prevention strategies that can be effectively applied in practice [16,17].

This study aligns with the initial phase of injury prevention frameworks, such as van Mechelen’s model [10], which emphasize the identification of injury incidence and patterns as a foundational step. Accordingly, we aimed to provide a descriptive analysis of training-related injury characteristics among youth field hockey players. This study aims to provide foundational data by examining the incidence, locations, and types of injuries occurring during training among South Korean youth field hockey players. Through an epidemiological analysis of training-related injuries, this study explores the key injury trends, with the goal of offering insights that could inform future efforts in injury prevention and management in this population.

## 2. Materials and Methods

### 2.1. Participants

This study included 374 field hockey players (236 males and 138 females) from middle and high school teams registered with the Korea Hockey Association in 2024. The participants’ ages ranged from 13 to 18 years. They agreed to participate after fully understanding that the results would be used for the study. This study was conducted based on the ethical principles of the Declaration of Helsinki. The survey was conducted from 1 October to 31 October 2024. Athletes voluntarily participated in the study after providing consent for the use of their personal information prior to the survey. One survey with insufficient responses was excluded, resulting in the analysis of 373 completed questionnaires. The number and physical characteristics of the participants in each group are presented in Table 1.

The participants were grouped by gender and school level (middle and high school) to examine potential differences in injury rates and experiences. The decision to group athletes by gender was based on the hypothesis that physiological differences, such as body composition, and hormonal factors, may influence injury risk. Additionally, grouping by school level was chosen to account for developmental differences in physical abilities and exposure to competitive play, as high school athletes typically have more advanced training and experience compared to middle school athletes. These factors were considered important for understanding how injury experiences may vary across different groups.

### 2.2. Survey Contents

The questionnaire was developed through expert meetings to investigate the causes of injuries among field hockey players and provide comprehensive baseline data for injury prevention and rehabilitation. The questionnaire was based on the International Olympic Committee (IOC) daily injury report form and the study by Chung et al. [18]. The survey gathered information on injury status (defined as being unable to participate in training or competition for ≥24 h within the past year), personal details (height, weight, age, and career), occurrence of injury during training, injury sites (upper limbs, trunk, lower limbs, head, etc.), injury tissues (skin, muscles, tendons, bones, ligaments, cartilage, joints, etc.), weekly training hours, and injury prevention strategies. Responses were allowed to be either singular or plural depending on the question, and participants were required to submit only one survey to avoid redundancy and ensure reliability.

### 2.3. Statistical Analysis

The data obtained in this study were analyzed using SPSS version 25.0 (IBM Corp., Amonk, NY, USA). The physical characteristics of the participants were presented as means (M) and standard deviations (SD). Chi-square (χ^2^) analysis was performed to assess differences in injury occurrence by gender and school level. Results on injury sites, tissues, and injury prevention strategies were presented as percentages (%) by gender and school level. The annual training hours were estimated using participants’ self-reported daily training hours and weekly training days. The injury incidence rate per 1000 training hours was calculated by dividing the number of injury occurrences by each individual’s annual training hours and multiplying the result by 1000. Additionally, we compared groups based on individual injury incidence rates. Since the assumption of normality was not satisfied, we applied the non-parametric Mann-Whitney U test. Furthermore, effect sizes (Cohen’s d and r) and 95% confidence intervals were calculated to provide a more comprehensive interpretation of the results. According to conventional benchmarks, Cohen’s d values of 0.2, 0.5, and 0.8 are considered small, medium, and large, respectively, while r values of 0.1, 0.3, and 0.5 indicate small, medium, and large effect sizes, respectively. Statistical significance was set at α = 0.05.

## 3. Results

### 3.1. Sports Injury Experiences by Gender and School Level

The analysis of sports injury experiences by gender revealed that 17.2% (n = 64) of male athletes had experienced injuries, while 10.2% (n = 38) of female athletes had reported similar experiences. However, this difference was not statistically significant. Additionally, an analysis based on school level indicated that 12.6% (n = 47) of middle school athletes and 14.7% (n = 55) of high school athletes had experienced sports injuries; however, this difference was also not statistically significant (Table 2).

### 3.2. Incidence Rate of Sports Injuries per 1000 Training Hours by Gender and School Level

Table 3 presents the incidence rates of sports injuries per 1000 training hours among field hockey players, stratified by gender and school level. The Mann–Whitney U test was conducted to compare injury rates per 1000 training hours between male and female athletes, as well as between middle and high school athletes. In terms of gender, male athletes (n = 236) had a mean rank of 185.73, while female athletes (n = 137) had a mean rank of 189.19. The Mann–Whitney U statistic was 15,866.00, with a Z value of −0.381 and a *p*-value of 0.703, indicating no statistically significant difference in injury rates between male and female athletes. Similarly, in terms of school level, middle school athletes (n = 183) had a mean rank of 186.01, and high school athletes (n = 190) had a mean rank of 187.95. The Mann–Whitney U statistic was 17,204.50, with a Z value of −0.221 and a *p*-value of 0.825, also indicating no statistically significant difference between the two groups. These findings suggest that neither gender nor school level significantly influences injury rates per 1000 training hours in this population, as both comparisons yielded *p*-values greater than 0.05, indicating that the observed differences in mean ranks were not statistically significant.

### 3.3. Analysis of Injury Sites by Gender and School Level

The analysis of injury sites by gender yielded the following results (Table 4). Among male athletes, the ankle was the most frequently injured site (19 cases, 18.8%), followed by the knee joint (16 cases, 15.8%) and the thigh (12 cases, 11.9%). Similarly, among female athletes, the ankle was the most commonly injured site (23 cases, 22.8%), followed by the wrist (12 cases, 11.9%) and the knee joint (10 cases, 9.9%). Additionally, the analysis of injury sites by school level indicated that, among middle school athletes, the ankle was the most frequently injured site (25 cases, 24.8%), followed by the wrist (11 cases, 10.9%), the hand (10 cases, 9.9%), and the knee joint (10 cases, 9.9%). Among high school athletes, the ankle (17 cases, 16.8%) and the knee joint (16 cases, 15.8%) were the primary injury sites, followed by the wrist (9 cases, 8.9%), the lower back (9 cases, 8.9%), and the thigh (8 cases, 7.9%).

### 3.4. Analysis of Injury Types by Gender and School Level

The analysis of injury types by gender revealed the following results (Table 5). Among male athletes, muscles were the most frequently injured tissue (30 cases, 30.0%), followed by ligaments (22 cases, 22.0%) and bones (20 cases, 20.0%). In contrast, among female athletes, ligaments were the most frequently injured tissue (20 cases, 20.0%), followed by muscles (18 cases, 18.0%) and tendons (8 cases, 8.0%). Additionally, the analysis of injury types by school level showed that, among middle school athletes, ligaments (20 cases, 20.0%) and muscles (19 cases, 19.0%) were the most commonly injured tissues, followed by bones (13 cases, 13.0%). Among high school athletes, muscles (29 cases, 29.0%) and ligaments (22 cases, 22.0%) were the most common injury types, followed by bones (14 cases, 14.0%).

### 3.5. Factors Considered Important for the Prevention of Sports Injuries

The analysis of factors considered important for the prevention of sports injuries yielded the following results (Table 6). Among male athletes, warm-up and cool-down exercises were identified as the most critical preventive measures (170 cases, 45.6%), followed by adequate rest (85 cases, 22.8%) and mental focus or stress management (37 cases, 9.9%). Similarly, among female athletes, warm-up and cool-down exercises (107 cases, 28.7%) were the most significant preventive measure, with adequate rest (37 cases, 9.9%) and mental focus or stress management (16 cases, 4.3%) ranking as secondary contributors. Regarding school level, middle school athletes most frequently cited warm-up and cool-down exercises (135 cases, 36.2%) as the primary preventive factor, followed by adequate rest (63 cases, 16.9%) and mental focus or stress management (32 cases, 8.6%). High school athletes also prioritized warm-up and cool-down exercises (142 cases, 38.1%) as the primary preventive measure, with adequate rest (59 cases, 15.8%) and mental focus or stress management (21 cases, 5.6%) ranking second and third, respectively.

## 4. Discussion

In open skill sports like field hockey, the ability of athletes to quickly react and adapt to unpredictable situations determines their skill level [19]. Due to recently adapted rules, field hockey has become a fast-paced game, where the ability to anticipate, adapt, and respond successfully relies on superior perceptual–cognitive factors [20]. Cognitive function consists of several sub-components such as perception, attention, and executive function [21], all of which contribute to successful skill and sports performance [22]. This ability to react quickly and accurately also plays an important role in injury prevention. Sports injuries are a critical factor in both enhancing and maintaining athletic performance, with prevention being essential for a successful athletic career [23]. Time lost due to injury impacts not only an athlete’s short-term participation but also negatively affects team performance and the outcomes of seasons or tournaments in the long term [24,25]. Therefore, injury prevention is a fundamental aspect of athlete management, playing a key role in ensuring both performance enhancement and sustained participation. This study aimed to investigate injury incidence, injury locations, and types of injuries among Korean youth field hockey players to identify the characteristics of sports injuries. The goal is to understand injury patterns and provide foundational data for developing effective injury prevention programs and strategies to improve athletic performance.

In this study, the Mann–Whitney U test was conducted to compare the sports injury rates per 1000 training hours between male and female athletes, as well as between middle and high school athletes. For gender, male athletes (n = 236) had a mean rank of 185.73, while female athletes (n = 137) had a mean rank of 189.19. The Mann–Whitney U statistic was 158,66.00, with a Z value of −0.381 and a *p*-value of 0.703, indicating no significant difference in injury rates between male and female athletes. Similarly, for school level, middle school athletes (n = 183) had a mean rank of 186.01, while high school athletes (n = 190) had a mean rank of 187.95. The Mann–Whitney U statistic for this comparison was 172,04.500, with a Z value of −0.221 and a *p*-value of 0.825, suggesting no significant difference between middle and high school athletes in terms of injury rates. Meanwhile, the average injury rate for male athletes was 0.46 injuries per 1000 training hours, and for female athletes, it was 0.60 injuries per 1000 training hours. For middle school athletes, the average injury rate was 0.58 injuries per 1000 training hours, while for high school athletes, it was 0.44 injuries per 1000 training hours. This is consistent with previous studies on Korean middle and high school fencing athletes, which reported higher injury rates among female athletes compared to male athletes and middle school athletes compared to high school athletes [18]. A study on handball players similarly found that female athletes (37 injuries, 69.8%) experienced more injuries than male athletes (16 injuries, 30.2%), and that middle and high school athletes (29.75 h) accumulated more training hours than university and adult athletes (22.70 h) [26]. In a similar study, the injury incidence during training for youth male basketball players in England (average age: 17.3 ± 0.9 years) was reported as 2.4 injuries per 1000 training hours [27], while in youth ice hockey, the injury incidence was approximately 1.2 injuries per 1000 training hours [28]. The injury rates in high-intensity sports like field hockey are similarly high, illustrating the relationship between training intensity and injury occurrence. These findings suggest that youth athletes with higher training volumes are more exposed to increased injury risks, highlighting the importance of regulating training intensity and implementing injury prevention programs.

In contrast, Theilen et al. reported a higher injury rate per game among male athletes (1.2 injuries per game) compared to female athletes (0.7 injuries per game) [29], indicating a discrepancy with the present findings. This difference may be attributed to variations in the factors influencing injury incidence, particularly the frequency and intensity of participation in games, which may have had a different impact in this study. However, the current findings support existing research suggesting that less mature and less technically developed athletes are more prone to injury. Faude et al. found that youth football players with less technical and physical maturity had higher injury rates [30], and that improvements in team size, training environments, and technical proficiency could help reduce injury risks [31,32]. The higher injury rate per training hour observed in female athletes may be influenced not only by physical differences but also by variations in training intensity and injury prevention awareness [17]. These results underscore the importance of adjusting training intensity and focusing on technical skill development to reduce injury rates [9,33] and emphasize the need for a balanced approach to technical skill development, physical conditioning, and training intensity in youth field hockey players.

An analysis of injury locations among Korean youth field hockey players revealed that the lower extremities, particularly the ankle (42 cases, 20.0%) and knee (26 cases, 12.0%), exhibited the highest injury rates. This finding is consistent with Furlong et al. (2018), who also reported the highest incidence of injuries in the foot and ankle region (6 cases in male athletes, 12 cases in female athletes) [16]. However, a study by Kim et al. on Korean national female field hockey players found that thigh (16 cases), ankle (13 cases), and knee (8 cases) injuries were the most prevalent among lower extremity injuries [34]. This discrepancy may be attributed to variations in training participation, intensity, and accumulated fatigue, particularly considering the higher incidence of thigh injuries among high school athletes compared to middle school athletes in the present study. Movement patterns specific to field hockey, such as body contact, evasion of the ball, rapid directional changes, and sudden acceleration and deceleration, contribute to higher injury rates [4,35].

Regarding upper extremity injuries, the wrist (20 cases, 9.5%) and hand (15 cases, 7.1%) showed the highest incidence rates, while lower back injuries were notably prevalent in the trunk region (17 cases, 8.1%). Previous studies have also documented high injury rates in the upper extremities, with hand and wrist injuries (87 cases, 11.14%) being the most common among NCAA female athletes [36], and hand and wrist injuries (20 cases, 3.9%) being the most frequent among high school athletes in the United States, followed by lower back injuries (38 cases, 7.5%) [14]. These injuries are commonly linked to the technique of gripping the stick, as well as tackles and set plays [37]. Moreover, performing dribbling maneuvers while in a bent posture has been shown to cause approximately 2.73 mm of spinal compression, increasing the likelihood of back injuries, which are among the most common in field hockey [38]. Additionally, female athletes were more than twice as likely as male athletes to be injured by the ball or stick. This suggests that improving technical skills, particularly in intercepting the ball, is crucial for injury prevention [16].

In this study, the analysis of injury types among youth field hockey players revealed that muscle injuries (48 cases, 27.9%) were the most prevalent, followed by ligament sprains/tears (42 cases, 24.4%) and bone fractures (27 cases, 15.7%). These findings align with epidemiological studies on the Korean female national field hockey team, where muscle injuries (36.9%) and ligament sprains/tears (30.4%) were also identified as the most common injury types [34]. Similarly, a study on sub-elite football players reported a high incidence of muscle injuries (429 cases, 41%) and ligament sprains/tears (270 cases, 26%) [39]. These results suggest that muscle and ligament injuries are common in high-intensity sports such as field hockey. Muscle injuries, in particular, are often caused by sudden directional changes, excessive tension, repetitive kicking and sprinting, and reduced flexibility, which are prevalent in sports such as football, basketball, field hockey, and tennis [40,41,42].

Furthermore, the use of artificial turf in field hockey warrants attention. Research has shown that in football, games played on artificial turf resulted in a 16% higher rate of lower extremity injuries compared to natural grass during the regular season [43]. Additionally, artificial turf has been found to double the incidence of ligament sprains compared to natural grass [44]. These findings emphasize that different sports may exhibit unique injury patterns and types, suggesting that injury prevention programs should focus on strengthening, balance, mobility, and sport-specific proprioceptive exercises tailored to the athlete’s level and the specific demands of the sport.

In this study, youth field hockey players most frequently identified warm-up and cool-down exercises (277 cases, 55.4%) and adequate rest (122 cases, 24.4%) as the most important factors for preventing sports injuries. These findings are consistent with studies on Korean youth fencing athletes and elite handball players, who also emphasized the importance of warm-up and cool-down exercises, as well as rest, as key strategies for injury prevention [18,26]. This suggests that elite athletes are well aware of the significance of these routines. For effective injury prevention, it is essential not only to raise athletes’ awareness but also to identify risk factors that may lead to injuries, such as low muscle strength, inadequate physical conditioning, fatigue, and excessive training, and to adjust training volume and intensity accordingly [45]. Furthermore, insufficient rest can have detrimental effects on physical and neurocognitive abilities, increase injury risk, and negatively impact sleep quality, highlighting the importance of ensuring adequate recovery [46,47]. In sports similar to field hockey, such as football, the implementation of the FIFA 11+ program has significantly reduced the incidence of non-contact injuries [48], with similar effects observed in basketball [49]. Additionally, long-term cognitive and perceptual task training has been recommended as it not only improves athletes’ motor skills and decision-making abilities but also positively impacts motor stability and enhances inter-joint coordination [50,51]. Therefore, it is crucial to systematically incorporate these components into warm-up programs for field hockey based on findings from various epidemiological studies. However, this study relied on self-reported injury data, and no medical verification was conducted. This limitation may introduce biases or inaccuracies in injury reporting. Future studies should consider including medical assessments for more accurate injury data.

## 5. Conclusions

This study aimed to analyze the injury incidence, locations, and types among youth field hockey players to identify key characteristics of sports injuries. The results indicated that muscle injuries, ligament sprains/tears, and bone fractures were the most common types of injuries. Athletes also recognized the importance of warm-up and cool-down exercises for injury prevention. Based on these findings, it is suggested that youth players may benefit from strength, balance, mobility, and proprioceptive training programs tailored to the specific demands of the sport. However, the study does not provide enough evidence to propose specific injury prevention strategies. Therefore, while this study highlights the need for a comprehensive approach to injury prevention, including scientific epidemiological investigations, training intensity regulation, skill development, and rest management, additional research is required to substantiate these recommendations. Future research should explore the relationship between biomechanical factors, reaction time assessments, and agility-based interventions in reducing injury incidence. Further studies are necessary to explore the effectiveness of tailored prevention programs based on biomechanical analysis and athlete response, with the goal of refining injury prevention strategies for youth field hockey players.

## Figures and Tables

**Table 1:** Physical characteristics of the participants.

**Group**	**Height (cm)**	**Weight (kg)**	**BMI (kg/m^2^)**	**Career (Years)**
Male (n = 236)	171.94 ± 7.16	65.20 ± 11.59	21.95 ± 2.99	2.59 ± 1.05
Female (n = 137)	160.50 ± 5.19	54.42 ± 8.17	21.10 ± 2.91	2.15 ± 1.04
Middle School (n = 183)	164.80 ± 7.75	57.49 ± 11.66	21.05 ± 3.26	1.72 ± 0.66
High School (n = 190)	170.56 ± 8.31	64.86 ± 10.52	22.20 ± 2.57	3.12 ± 0.92
Total (n = 373)	167.74 ± 8.53	61.24 ± 11.67	21.64 ± 2.98	2.43 ± 1.06

BMI: Body Mass Index.

**Table 2:** Result of sports injury occurrence during training by gender and school level.

Group	N(%)			χ^2^ (*p*)	Effect Size (Cohen’s d)	95% CI
	IEG	IFG	Total			
Male	64(17.2)	172(46.1)	236(63.3)	0.017	−0.12	−0.38–0.14
Female	38(10.2)	99(26.5)	137(36.7)	(0.897)		
Middle School	47(12.6)	136(36.5)	183(49.1)	0.500	0.12	−0.11–0.36
High School	55(14.7)	135(36.2)	190(50.9)	(0.480)		
Total	102(27.3)	271(72.7)	373(100)			

IEG: Injury Experience Group, IFG: Injury Free Group, Cohen’s d: Effect size calculated using the difference between group means, interpreted as small (0.2), medium (0.5), or large (0.8) effect, CI: Confidence Interval.

**Table 3:** Sports injury rates per 1000 training hours by gender and school level.

Group	Injuries/1000 h Mean Rank	U	Z	*p*	Effect Size (r)
Male (n = 236)	185.73	158,66.00	−0.381	0.703	−0.02
Female (n = 137)	189.19				
Middle School (n = 183)	186.01	172,04.500	−0.221	0.825	−0.01
High School (n = 190)	187.95				
Total (n = 373)	0.51 ± 1.14				

r: Effect size calculated from Z statistic, interpreted as small (0.1), medium (0.3), or large (0.5) effect.

**Table 4:** Analysis of injury sites in sports based on gender and school level.

Injury Sites	N(%)				Total
	Gender		School Level		
	Male	Female	Middle School	High School	
Head	3(3.0)	3(3.0)	1(1.0)	5(5.0)	6(2.9)
Face	7(6.9)	3(3.0)	5(5.0)	5(5.0)	10(4.8)
Neck	NA(0.0)	1(2.6)	1(1.0)	NA(0.0)	1(0.5)
Sternum	1(1.0)	NA(0.0)	NA(0.0)	1(1.0)	1(0.5)
Ribs	2(2.0)	NA(0.0)	NA(0.0)	2(2.0)	2(1.0)
Back	4(4.0)	1(1.0)	3(3.0)	2(2.0)	5(2.4)
Lower Back	10(9.9)	7(6.9)	8(7.9)	9(8.9)	17(8.1)
Hip	2(2.0)	2(2.0)	3(3.0)	1(1.0)	4(1.9)
Shoulder	8(7.9)	2(2.0)	4(4.0)	6(5.9)	10(4.8)
Upper Arm	1(1.0)	NA(0.0)	NA(0.0)	1(1.0)	1(0.5)
Elbow	2(2.0)	2(2.0)	2(2.0)	2(2.0)	4(1.9)
Lower Arm	3(3.0)	1(1.0)	2(2.0)	2(2.0)	4(1.9)
Wrist	8(7.9)	12(11.9)	11(10.9)	9(8.9)	20(9.5)
Hand	8(7.9)	7(6.9)	10(9.9)	5(5.0)	15(7.1)
Pelvis	4(4.0)	4(4.0)	5(5.0)	3(3.0)	8(3.8)
Thigh	12(11.9)	5(5.0)	9(8.9)	8(7.9)	17(8.1)
Knee Joint	16(15.8)	10(9.9)	10(9.9)	16(15.8)	26(12.4)
Calf	5(5.0)	4(4.0)	4(4.0)	5(5.0)	9(4.3)
Ankle	19(18.8)	23(22.8)	25(24.8)	17(16.8)	42(20.0)
Foot	5(5.0)	3(3.0)	3(3.0)	5(5.0)	8(3.8)
Total	120(63.4)	90(36.6)	106(50.4)	104(49.5)	210(100)

**Table 5:** Analysis of injury types in sports based on gender and school level.

Injury Types	N(%)				
	Gender		School Level		Total
	Male	Female	Middle School	High School	
Skin	8(8.0)	7(7.0)	8(8.0)	7(7.0)	15(8.7)
Muscle	30(30.0)	18(18.0)	19(19.0)	29(29.0)	48(27.9)
Tendon (Ligament)	9(9.0)	8(8.0)	9(9.0)	8(8.0)	17(9.9)
Bone	20(20.0)	7(7.0)	13(13.0)	14(14.0)	27(15.7)
Ligament	22(22.0)	20(20.0)	20(20.0)	22(22.0)	42(24.4)
Cartilage	3(3.0)	3(3.0)	4(4.0)	2(2.0)	6(3.5)
Meniscus	1(1.0)	2(2.0)	2(2.0)	1(1.0)	3(1.7)
Joint	4(4.0)	6(6.0)	6(6.0)	4(4.0)	10(5.8)
Other	2(2.0)	2(2.0)	1(1.0)	3(3.0)	4(2.3)
Total	99(57.6)	73(42.4)	82(47.6)	90(52.3)	172(100)

**Table 6:** Analysis of key factors for preventing sports injuries.

Key Factors	N(%)				
	Gender		School level		Total
	Male	Female	Middle School	High School	
Warm-up and cool-down exercises	170(45.6)	107(28.7)	135(36.2)	142(38.1)	277(55.4)
Adequate rest	85(22.8)	37(9.9)	63(16.9)	59(15.8)	122(24.4)
Tension or mental focus	37(9.9)	16(4.3)	32(8.6)	21(5.6)	53(10.6)
Improvement of physical conditioning	20(5.4)	7(1.9)	17(4.6)	10(2.7)	27(5.4)
Reduction of practice time	3(0.8)	4(1.1)	5(1.3)	2(0.5)	7(1.4)
Improvement of coaching methods	3(0.8)	2(0.5)	2(0.5)	3(0.8)	5(1.0)
Improvement of facilities and equipment	5(1.3)	3(0.8)	6(1.6)	2(0.5)	8(1.6)
Improvement of competition methods	1(0.3)	NA(0.0)	NA(0.0)	1(0.5)	1(0.2)
Total	324(63.3)	176(36.7)	260(49.1)	240(50.9)	500(100)

## Data Availability

The data presented in this study are available on request from the corresponding author.
